# Assessment of the Efficacy and Safety of a Dual-Wavelength Diode Laser System for the Treatment of Vulvovaginal Atrophy in Women Without a History of Breast Cancer and in Patients with a History of Breast Cancer

**DOI:** 10.3390/jcm14030801

**Published:** 2025-01-26

**Authors:** Gaetano Perrini, Silvia Actis, Margherita Giorgi, Francesca Accomasso, Carola Minella, Cristina Fava, Giorgia Bisconte, Giovanni De Rosa, Annamaria Ferrero, Valentina Elisabetta Bounous

**Affiliations:** 1 Gynecology and Obstetrics Unit, Mauriziano Umberto I Hospital, Largo Turati 62, 10128 Turin, Italy; gperrini@mauriziano.it; 2 Gynecology and Obstetrics Unit, Mauriziano Umberto I Hospital, Department of Surgical Sciences, University of Turin, 10128 Turin, Italy; margherita.giorgi@unito.it (M.G.); francesca.accomasso@unito.it (F.A.); carola.minella@unito.it (C.M.); giorgia.bisconte@unito.it (G.B.); annamaria.ferrero@unito.it (A.F.); valentinaelisabetta.bounous@unito.it (V.E.B.); 3 Pathological Anatomy Unit, Mauriziano Umberto I Hospital, Largo Turati 62, 10128 Turin, Italy; cfava@mauriziano.it (C.F.); gderosa@mauriziano.it (G.D.R.)

**Keywords:** laser, vulvovaginal atrophy (VVA), genitourinary syndrome of menopause (GSM), menopause, breast cancer

## Abstract

**Background/Objectives**: Vaginal laser therapy is a promising treatment for menopausal vulvovaginal atrophy (VVA). This study evaluates the efficacy of a dual-wavelength diode laser (980 + 1470 nm) in treating VVA. **Methods**: Thirty postmenopausal patients with moderate-to-severe VVA were recruited, and twenty-nine completed the study. Patients underwent a screening visit (T0); three laser sessions at 4-week intervals (T1, T2, and T3); and a follow-up visit 4 weeks after the last treatment (T4). At T0 and T4, the Schiller test and Vaginal Maturation Index (VMI) were performed; sexual function was assessed through the Female Sexual Function Index (FSFI) and the Female Sexual Distress Scale-Revised (FSDS-R). At each visit, the Vaginal Health Index (VHI) and the Visual Analog Scale (VAS) for dyspareunia were administered. Potential side effects were investigated, and the VAS for the pain associated with the procedure was assessed. Data analysis included the overall group and two subgroups: women with (group A) and without (group B) a history of breast cancer (BC). **Results**: Significant improvements in the VHI and reduced dyspareunia were observed at T4 compared to T0 in all groups. The improvement was already seen after the first procedure, with further improvement after the other procedures, being persistent at the 1-month follow-up. The Schiller test showed significant improvements from T0 to T4 in all groups. The VMI showed a significant improvement from T0 to T4 in the overall group and group B. The FSFI questionnaires showed a significant improvement in all areas for the whole population, whereas the FDSF-R questionnaire showed an improvement only in the overall group. Procedural pain was low (mean VAS 1.6), and no side effects were reported. **Conclusions**: The dual-wavelength diode laser is an effective and safe option for the treatment of VVA in patients with and without a history of BC.

## 1. Introduction

The genitourinary syndrome of menopause (GSM) includes all the genital, urinary, and sexual signs and symptoms associated with the hormonal decline that occurs during menopause [1]. The prevalence of GSM is approximately 50% in menopausal women, although it is estimated that the syndrome is underdiagnosed and consequently undertreated [2]. The genital symptoms of GSM are described as vulvovaginal atrophy (VVA) and include vaginal dryness, dyspareunia, lack of lubrication, irritation during physical activity, and post-coital bleeding [3]. Unlike vasomotor symptoms, VVA is chronic and rarely resolves if left untreated.

The onset of this syndrome is due to the changes that occur in the squamous epithelium of the vagina because of the decrease in endogenous estrogen. During menopause, the superficial cells with a high glycogen content reduce, vaginal pH increases, elastin decreases, connective tissue increases, collagen hyalinization occurs, and there is a progressive loss of vascularization [4].

Various studies have shown that VVA has a significant impact on many aspects of a woman’s life, including exercise, work, sexual activity, relationship with a partner, enjoyment of life, social activities, and travel [3].

Treatments available for VVA include non-hormonal moisturizing creams or lubricants, vaginal estrogens, hormone replacement therapy, ospemifene, pelvic floor rehabilitation, and, in an experimental context, vaginal lasers [5,6,7]. Vaginal lasers are a mechanical treatment [8,9,10], and it is a valid alternative for patients who are contraindicated to or do not wish to take hormonal therapies [11].

At present, most studies of laser treatment of VVA have been carried out with two lasers: the fractional CO_2_ laser and the Erbium-YAG laser. Many studies have been published on laser therapy, showing a significant improvement in the symptoms of VVA, highlighting the efficacy and safety of this treatment and a greater durability of the benefits obtained compared to other treatments.

The diode laser is already widely used in hysteroscopic surgery to perform polypectomies and myomectomies [12,13]. As the physical principle of the diode laser is similar to that of CO_2_ or Erbium lasers, its use in the context of VVA is expected to be beneficial. Data on the use of diode lasers in the treatment of VVA are limited compared to the previously mentioned methods but appear promising in terms of benefits for both vaginal histological features and perceived symptoms [14,15].

This study aims to evaluate the performance of dual-wavelength diode laser treatment (Leonardo Dual ^®^, Biolitec, Jena, Germany) in the treatment of menopausal VVA.

## 2. Materials and Methods

The study was conducted at the Gynecology and Obstetrics Unit of the Mauriziano Umberto I Hospital in Turin. The Institutional Ethics Committee of Mauriziano Umberto I Hospital approved the study protocol (Prot. No. 20/2023 v1.0; 17 November 2022).

A total of thirty postmenopausal women were included in the study, with a primary aim to evaluate the effects of diode laser treatment for moderate-to-severe vulvovaginal atrophy (VVA) symptoms. Subgroup analyses were planned to assess outcomes specifically in women with and without a history of breast cancer, and this study employed a non-randomized observational design. The sample size (*n* = 30) was determined based on feasibility and prior studies indicating significant improvements in the VVA outcomes with sample sizes ranging between 20 and 40 patients.

The inclusion criteria were:

- Postmenopausal women (defined as >12 months since last menstrual period or 6 months of amenorrhea with serum follicle-stimulating hormone (FSH) levels > 40 mIU/mL or post-adnexectomy);
- Women between the ages of 45 and 70;
- Acceptance of the study and signing of informed consent;
- Moderate-to-severe VVA assessed by the VHI;
- Negative cervical smear test within the last 12 months;
- Clinical absence of ongoing inflammatory or infectious conditions;
- Absence of concurrent active neoplasia.

The exclusion criteria were:

- Positive cervical smear test or ASCUS (Atypical Squamous Cells of Undetermined Significance);
- Ongoing inflammatory or infectious diseases;
- Non-menopausal patients (<12 months since last menstrual period or 6 months of amenorrhea with serum follicle-stimulating hormone (FSH) levels < 40 mIU/mL);
- Patients taking systemic or local hormone therapy;
- Coagulation disorders;
- Bleeding of unknown origin.

To evaluate AVV before and after diode laser treatment, differences and the variability of differences in the following parameters were evaluated before the first laser session (T0) and 4 weeks after the last procedure (T4):

- Visual inspection of the vagina and the cervix with Lugol’s iodine (Schiller test), which was graded by an experienced colposcopist from 1 (much reduced Lugol’s stain) to 3 (normal Lugol’s stain).
- Perceived symptoms by the Female Sexual Function Index (FSFI) [16] and Female Sexual Distress Scale-Revised (FSDS-R) [17] were used to assess the changes in the perception of dyspareunia and sexual wellbeing (Supplementary File S1 and S2).
- Cytology by the Vaginal Maturation Index (VMI) indicates the degree of maturation by measuring the percentages of superficial, intermediate, and parabasal cells. The Maturity Value (MV) is calculated using the following formula: MV = (0 × % parabasal cells) + (0.5 × % intermediate cells) + (1.0 × % superficial cells), and that was graded from 1 (mild atrophy) to 4 (severe atrophy) by an experienced pathologist [18].

To assess AVV before and after diode laser treatment, the differences and the variability of differences in the following parameters were assessed before each session (T0/1, T2, and T3) and 4 weeks after the last procedure (T4):

- Vaginal Health Index (VHI), a tool that allows the presence and severity of AVV to be defined by assessing five parameters (vaginal elasticity, vaginal secretion, pH, epithelial mucosa, and vaginal hydration), which are given individual scores to calculate the final score. The total score can vary from 5 to 25. If the score is <15, the vagina is considered atrophic [19].
- Visual Analog Scale (VAS) to evaluate dyspareunia. The VAS rates from 0, none to 10, maximum discomfort.

The dual-wavelength 980 nm + 1470 nm laser system (Leonardo Dual ^®^, Biolitec, Germany) is used in this study. The dual-wavelength diode laser not only affects water molecules but is also absorbed by hemoglobin, which has a superior hemostatic effect. Lateral fiber optic delivery is used in this trial, which allows for more gentle and painless protocols for patients compared to the radial fiber optic delivery used in previous diode laser studies.

The trial was conducted as follows:

- T0 (First visit): The informed consent was signed; if no negative cervical smear test was available in the previous 12 months, a cervical smear test was performed. The VMI, Schiller vaginal test, and VHI were performed, and VAS for dyspareunia, FSFI, and FSDS-R questionnaires were collected.
- T1 (Second visit): The first vaginal laser procedure was administered.
- T2 (Third visit, scheduled 4 weeks after T1): The second vaginal laser procedure was administered after the VHI and VAS assessments for dyspareunia.
- T3 (Fourth visit, scheduled 4 weeks after T2): The third vaginal laser procedure was administered after the VHI and VAS assessments for dyspareunia.
- T4 (Fifth visit, scheduled 4 weeks after the last treatment): The VMI, Schiller vaginal test, and VHI were performed, and VAS for dyspareunia, FSFI, and FSDS-R questionnaires were administered.

**Figure 1:**
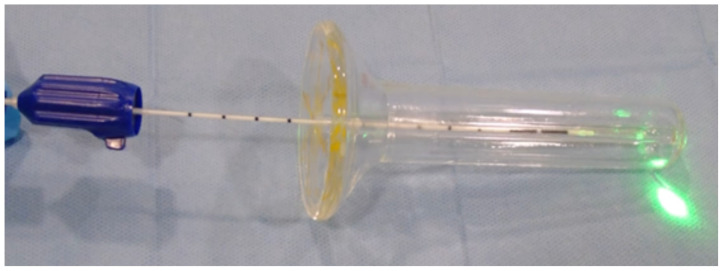
Applicator and optical fiber for laser delivery.

The procedure is carried out as follows: The patient is placed in the gynecological position, and the applicator is lubricated and inserted into the vaginal canal. The laser is set to pulsed mode (2 pulses) with a duration of 500 ms and a pause of 500 ms. The 7-watt laser is activated. The optical fiber is connected to the Leonardo dual laser, and the fiber is inserted inside the applicator to the bottom of the canal (Figure 1). Two pulses are delivered at the 2 o’clock, two pulses at the 4 o’clock, two pulses at the 8 o’clock, and two pulses at the 10 o’clock position. The fiber is withdrawn 1 cm, and the pulses are delivered at the 4 o’clock, 8 o’clock, and 10 o’clock positions, and the procedure is repeated for each centimeter of the vaginal canal.

### Statistical Analysis

The study sample is described at baseline with a median and interquartile range for the continuous variables and absolute frequency and percentage for the categorical variables. The statistical software IBM SPSS (1.0.0.1213) was used for analysis. The normality of the sample data was assessed using the Shapiro–Wilk test. In the case of variables with normally distributed data and repeated measurements, the statistical test used was ANOVA with repeated measures. If the data were not normally distributed, the non-parametric Friedman One-Way Repeated Measure Analysis of Variance by Ranks test was employed.

When repeated measures tests showed significant results (*p* < 0.05), post hoc tests were conducted to assess pairwise differences between groups or time points. To account for the increased risk of a type I error due to multiple comparisons, a Bonferroni correction was applied. Specifically, for comparisons across 4 variables and 6 time points, the adjusted significance threshold was set at *p* < 0.0083 (0.05/6).

For non-normal data, the Wilcoxon signed-rank test was used for pairwise comparisons of non-independent samples. For normally distributed data, pairwise comparisons were performed using the paired *t*-test.

If measurements were taken only at two time points (e.g., T0 and T4), changes over time were assessed using a significance level of *p* < 0.05. This distinction was made to ensure consistency in statistical interpretation, depending on the number of measurements performed.

The choice of statistical methods and corrections for multiple comparisons reflects the study’s objective to maintain robustness and reliability in the analysis while minimizing the likelihood of spurious findings.

## 3. Results

### 3.1. Study Population

Thirty patients were initially included in this study. Twenty-nine patients completed all three treatments and the follow-up visit 4 weeks after the last treatment, as one patient was diagnosed with breast cancer during the procedures and dropped out of the study to undergo oncological treatment.

The mean age of the study sample was 59.0 years old (S.D. 3.7), and the average age of menopause in our sample was 49.8 years old (S.D. 3.7). Of the 29 patients who completed the study, 13 had a history of breast cancer, constituting 45% of the sample (Group A). The remaining 16 patients (Group B) had no history of cancer.

### 3.2. Analysis of the Total Sample

**Figure 2:**
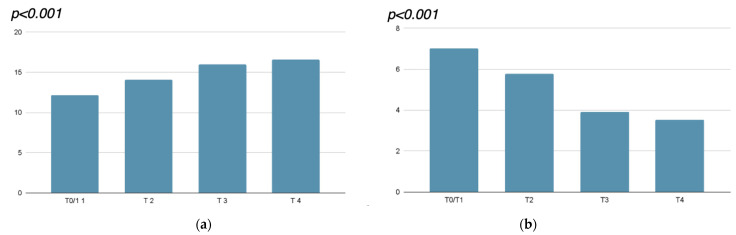
(**a**) VHI (Vaginal Health Index) and (**b**) VAS (Visual Analog Scale) for dyspareunia mean values at T0/T1, T2, T3, and T4.

A significant improvement in the VHI scores has emerged after each procedure, with the minimum VHI score for atrophy (15) being exceeded after the second session. A decrease in dyspareunia reported by the patients was also shown, as assessed by the VAS (Table 1 and Figure 2).

The VHI improved from T0 to T4, and the improvement was already seen after the first procedure, with further improvement after the other procedures, maintaining the effect at the 1-month follow-up (VHI: T0–T2, *p* < 0.001; T0–T3, *p* < 0.001; T0–T4, *p* < 0.001; T2–T3, *p* < 0.001; T2–T4, *p* < 0.001; T3–T4, *p* = 0.01. Note: *p*-values represent significance levels obtained from repeated-measures ANOVA. Where applicable, paired *t*-tests and Wilcoxon signed-rank tests were used depending on normality. To control for type I error, Bonferroni correction was utilized (0.05/6 = 0.0083 = alpha for four variables and 6 comparisons)).

The VAS scores for dyspareunia improved between T0 and T4. VAS reduction was already seen after the first procedure, with further improvement after the other procedures, maintaining the effect at the 1-month follow-up (VAS: T0–T2, *p* < 0.001; T0–T3, *p* < 0.001; T0–T4, *p* < 0.001; T2–T3, *p* < 0.001; T2–T4, *p* < 0.001; T3–T4, *p* = 0.228).

**Figure 3:**
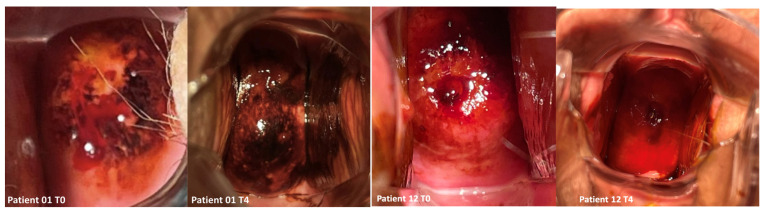
Schiller test was performed before any procedure (T0) and 4 weeks after the last laser treatment (T4).

The Schiller test, graded by an experienced colposcopist from 1 (much reduced Lugol’s stain) to 3 (normal Lugol’s stain), showed a significant improvement from T0 to T4 (*p* = 0.001) (Figure 3 and Table 2), proving a decreased vaginal atrophy.

**Figure 4:**
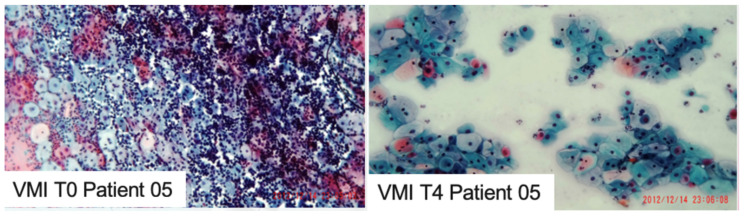
Vaginal Maturation Index (VMI) was performed before any procedure (T0) and 4 weeks after the last laser treatment (T4).

Cytology by the VMI, graded by an experienced pathologist from 1 (mild atrophy) to 4 (severe atrophy), showed a significant improvement from T0 to T4 (*p* = 0.007) (Figure 4 and Table 2).

Perceived symptom scores evaluated through the FSFI and FDS-R questionnaires showed a significant improvement in all areas (*p* < 0.001 for FSFI; *p* = 0.006 for FDS-R).

The mean VAS for procedural pain evaluated at the end of the three procedures was low (1.6).

### 3.3. Analyses of the Subgroups (Women with a History of Breast Cancer (BC) and Women with No History of BC)

The same analyses performed on the whole group were then performed on the two subgroups of women with a history of previous BC (Group A) and on the subgroup of patients with no history of oncological disease (Group B).

Significant improvements in the VHI and VAS scores were seen in both subgroups of patients with and without a history of BC (Table 3).

The VHI scores improved from T0 to T4, both in patients with (Group A: VHI: T0–T2, *p* = 0.002; T0–T3, *p* = 0.001; T0–T4, *p* = 0.001; T2–T3, *p* = 0.002; T2–T4, *p* = 0.001; T3–T4, *p* = 0.080) and without BC history (Group B: VHI: T0–T2, *p* = 0.001; T0–T3, *p* < 0.001; T0–T4, *p* < 0.001; T2–T3, *p* = 0.001; T2–T4, *p* = 0.001; T3–T4, *p* = 0.094) (Note: *p*-values represent significance levels obtained from repeated-measures ANOVA. Where applicable, paired *t*-tests and Wilcoxon signed-rank tests were used depending on normality. To control for type I error, Bonferroni correction was utilized (0.05/6 = 0.0083 = alpha for four variables and six comparisons)).

Improvement in the VAS scores for dyspareunia from T0 to T4 was confirmed in both groups (Group A: VAS T0–T4, *p* = 0.001; Group B: VAS T0–T4, *p* = 0.001), whereas, in patients without a history of BC, an improvement was shown after each procedure (VAS: T0–T2, *p* = 0.004; T0–T3, *p* = 0.001; T2–T3, *p* = 0.002; T2–T4, *p* = 0.003; T3–T4, *p* = 0.458; alpha = 0.0083), it appears that more than one treatment is required in patients with a BC history to achieve a significant improvement in pain, as assessed by the VAS (VAS: T0–T2, *p* = 0.011; T0–T3, *p* = 0.003; T2–T3, *p* = 0.030; T2–T4, *p* = 0.007; T3–T4, *p* = 0.324; alpha = 0.0083).

Analysis of the Schiller test showed a significant improvement from T0 to T4 in both groups of patients with (*p* = 0.020) and without BC history (*p* = 0.011) (Table 4).

Cytology by the VMI, graded from 1 (mild atrophy) to 4 (severe atrophy), showed a significant improvement from T0/1 to T4 in patients without BC history (*p* = 0.016) (Table 4).

Perceived symptom scores evaluated through the FSFI questionnaires showed a significant improvement in all areas for both groups of patients (Group A *p* = 0.007; Group B *p* = 0.027). No significant differences were seen in the FDSF-R questionnaires (Table 4).

## 4. Discussion

The main goal in treating GSM is to alleviate symptoms. Treatment for women with GSM can be approached step by step, depending on symptom severity. Initial treatments for milder symptoms involve using non-hormonal vulvar and vaginal lubricants during sexual activity, along with regular use of long-acting vaginal moisturizers. Prescription options include low-dose vaginal estrogens, vaginal DHEA inserts, and oral ospemifene. For women experiencing moderate-to-severe dyspareunia due to GSM, along with concurrent vasomotor symptoms, transdermal and oral hormone therapy are effective options. Regular therapy is typically necessary, as symptoms are likely to return if treatment is stopped [20], and unlike vasomotor symptoms, GSM tends to worsen over time [3]. Low-dose vaginal estrogen therapy (ET) should be used with caution in women with estrogen-dependent cancer, and some patients refuse or do not respond to local hormone treatment. It is therefore important to validate non-hormonal methods, among which, lasers could be considered in both oncological and non-oncological patients, with the lasers most used in clinical trials to date being CO_2_ and Erbium-YAG lasers [21].

The mechanism of action of laser therapy is that fractional beams of light create small wounds in the epithelium and lamina propria, which then lead to the stimulation of collagen, remodeling, and regeneration. It is also thought to increase blood flow to the area, improving tissue quality [22].

Studies on laser treatment were initially performed evaluating the effectiveness of CO_2_ vaginal lasers [23,24]. A 2022 meta-analysis on a total of 1152 patients from 25 studies on the effects of CO_2_ laser therapy on GSM symptoms assessed through subjective or objective efficacy measurement methods showed a significant reduction in VVA and/or GSM symptoms (dryness, dyspareunia, itching, burning, and dysuria); FSFI; and Vaginal Maturation Value (VMV) scores. Moreover, the CO_2_ laser application showed a beneficial safety profile, and no major adverse events were reported [10]. In more recent years, the non-ablative vaginal Erbium-YAG laser, has also been tested, showing a significant improvement in GSM symptoms and cytology [25,26,27,28,29].

A diode laser is a non-ablative laser that causes controlled thermal damage primarily targeting the lower connective tissue, thereby prompting collagen production while preserving the epithelium [15].

In our study, we observed a significant improvement in all VVA parameters following three sessions of a diode laser. An improvement in the VHI and a decrease in dyspareunia were observed, and the improvement was already seen after the first procedure, with further improvement after the other procedures, while still present at the 1-month follow-up. Atrophy evaluated by an expert pathologist by the Schiller test and cytology by the VMI showed a significant improvement as well. Sexual function, assessed by the FSFI questionnaires, increased significantly.

To our knowledge, non-ablative diode lasers are promising yet underrepresented in clinical trials that evaluate the effect of lasers on VVA symptoms. In a recent study by Barba M. et al. [15] on 26 patients who underwent non-ablative diode laser treatment with the same scheme as the one used in our study, the intensity of the VVA symptoms was evaluated through the VHI; the Patient Global Impression of Improvement (PGI-I) questionnaire; and VAS for vaginal burning, vaginal itching, vaginal dryness, dyspareunia, and dysuria, and the sexual function was evaluated with the FSFI-19 questionnaire. All areas analyzed in the study were found to improve after vaginal diode laser therapy, and no adverse events were reported in this study after laser treatment either. Even in our study, although with a short follow-up, no side effects occurred, and the mean VAS for procedural pain was low.

However, cytology and colposcopy data were not analyzed in the study by Barba M. et al. [15], and they did not include subgroups of patients with a history of cancer in their analysis, as they constituted a small sample of their population.

Following recovery from cancer surgery, chemotherapy, and radiation, the prognosis can be excellent, and a return to full health can often be expected, and yet, there may have been devastating changes to sexual function owing to the cancer treatment [30]. The incidence of sexual dysfunctions in women with cancer undergoing different cancer treatments ranges from 30% to 80%, while the risk of developing sexual dysfunction increases 2.7- and 3.5-fold in women with cervical and breast cancer, respectively [31]. Women with BC experience a greater impact of vaginal symptoms on emotional wellbeing, sexual functioning, and self-concept/body image than healthy controls [32].

CO_2_ and Erbium-YAG lasers, as non-hormonal treatment options, have shown promise in improving VVA symptoms, particularly in BC survivors, as evidenced by pilot studies, prospective open cohort studies, and retrospective cohort studies that reported significant clinical improvements in VVA symptoms (measured by the FSFI, VHI, and VAS) without major side effects during short-term follow-up [33,34]. Recent research further highlights the potential of non-ablative vaginal lasers for patients unable to undergo hormonal treatments, reaffirming their efficacy and safety [34].

In our study, we included BC survivors who experienced a significant improvement in VVA, with an improvement in all parameters and a mean overcome of the VHI threshold.

Our protocol utilized three sessions with 4-week intervals, designed to assess sustained efficacy and consistent with schedules commonly employed for other laser treatments. While previous studies on breast cancer survivors utilizing shorter intervals (e.g., 15–20 days) have demonstrated rapid symptom relief [34], our findings at the 4-week follow-up complement those of Lubián-López et al. [34], who reported sustained improvements lasting up to 6 months post-treatment. These results suggest that early-phase improvements can persist over extended follow-up periods, underscoring the potential long-term benefits of this treatment approach.

The limitations of this study include the small sample size, the lack of a control group, and the short follow-up. Our follow-up period was set at 4 weeks post-treatment to standardize comparisons across visits and minimize dropouts. Potential biases, such as selection and reporting biases, were carefully considered in this study. To minimize the selection bias, clear and stringent inclusion and exclusion criteria were established, ensuring that the study population was representative of the target group. Patients were screened rigorously based on these criteria, and informed consent was obtained to enhance transparency. Reporting bias was mitigated by using validated and standardized tools, such as the Female Sexual Function Index (FSFI), Vaginal Maturation Index (VMI), and Schiller test, which provide objective and consistent measures of outcomes. These measures aim to enhance the reliability and validity of the study findings.

## 5. Conclusions

In our study, the dual-wavelength diode laser is an effective, safe, and well-tolerated option for the treatment of VVA in patients with and without a history of breast cancer. Breast cancer survivors represent a population at high risk of disabling and treatment-resistant vulvovaginal symptoms. This condition requires special therapeutic attention, and the diode laser seems a promising option. Our findings align with the growing body of evidence supporting diode lasers, suggesting their potential. However, larger randomized trials with extended follow-ups are needed to validate this method further and establish optimal treatment protocols and follow-up strategies.

## Figures and Tables

**Table 1:** Mean and standard deviation of measurements taken before the procedures (T0/T1), before each treatment (T2 and T3), and 4 weeks after the last treatment (T4). VHI: Vaginal Health Index; VAS: Visual Analog Scale for dyspareunia.

	T0/1	T2	T3	T4	*p*
VHI	12.17 (1.47)	14.07 (1.46)	16.03 (1.90)	16.62 (1.95)	<0.001
VAS	7.03 (2.06)	5.79 (2.24)	3.93 (2.58)	3.52 (2.61)	<0.001

**Table 2:** Mean and standard deviation of measurements taken before the procedures (T0) and 4 weeks after the last treatment (T4).

	T0	T4	*p*
Shiller’s test	1.93 (0.80)	2.45 (0.51)	0.001
VMI	2.69 (1.28)	2.1 (1.26)	0.007
FSFI	13.12 (8.7)	18.16 (9.60)	<0.001
FDS-R	25.21 (15.82)	17.76 (13.68)	0.006

**Table 3:** Mean and standard deviation of measurements taken before the procedures (T0/T1), before each treatment (T2 and T3), and 4 weeks after the last treatment (T4) in patients (a) with breast cancer history (Group A) and (b) without a history of breast cancer (Group B).

(**a**)					
	**T0/1**	**T2**	**T3**	**T4**	***p***
VHI	12.00 (1.41)	13.62 (1.19)	15.38 (1.80)	15.92 (1.44)	<0.001
VAS	7.77 (1.79)	6.46 (1.94)	4.62 (2.26)	4.00 (2.48)	<0.001
(**b**)					
	**T0/1**	**T2**	**T3**	**T4**	***p***
VHI	12.31 (1.54)	14.44 (1.59)	16.56 (1.86)	17.19 (1.167)	<0.001
VAS	6.44 (2.13)	5.25 (2.38)	3.38 (2.75)	3.13 (2.73)	<0.001

**Table 4:** Mean and standard deviation of measurements taken before the procedures (T0/T1) and 4 weeks after the last treatment (T4). (a) With breast cancer history (Group A) and (b) without breast cancer history (Group B).

(**a**)			
	**T0**	**T4**	***p***
Shiller’s test	1.86 (0.77)	2.31 (0.48)	0.020
VMI	2.93 (1.07)	2.54 (1.33)	0.141
FSFI	10.56 (7.35)	18.17 (7.80)	0.007
FDS-R	28.07 (18.41)	20.79 (15.78)	0.083
(**b**)			
	**T0**	**T4**	***p***
Shiller’s test	2.06 (0.85)	2.56 (0.51)	0.011
VMI	2.44 (1.41)	1.75 (1.12)	0.016
FSFI	14.79 (9.56)	17.80 (11.00)	0.027
FDS-R	21.44 (13.36)	15.13 (10.85)	0.137

## Data Availability

The data presented in this study are available from the corresponding author on reasonable request. The data are not publicly available due to privacy policies.

## Supplementary Materials

The following supporting information can be downloaded at https://www.mdpi.com/article/10.3390/jcm14030801/s1.
