# Supplementary material for: Assessment of the Efficacy and Safety of a Dual-Wavelength Diode Laser System for the Treatment of Vulvovaginal Atrophy in Women Without a History of Breast Cancer and in Patients with a History of Breast Cancer
Source: J Clin Med. 2025 Jan 26;14(3):801. doi: 10.3390/jcm14030801 (PMC11818461; doi:10.3390/jcm14030801)
Supplement: Supplementary file 1 [file jcm-14-00801-s001.zip › Suppl file s2.pdf]

Machine Translated by Google

Hospital Company
Mauritian Order of Turin

Registered office: Via Magellano, 1 - 10128 TURIN - Tel. +39 011.508.1111 - www.mauriziano.it - VAT/Tax Code 09059340019

MATERNAL AND CHILD CARE DEPARTMENT
SCDU GYNECOLOGY and OBSTETRICS Director:
Prof. N. Biglia
Largo Turati, 62 Turin
Telephone: 011/508.2682 Fax:
011/508.2265- 2683
e-mail: ostetricia@mauriziano.it
gynecology@mauriziano.it

UNIVERSITÀ DEGLI STUDI DI TORINO
ALMA UNIVERSITAS TAURINENSIS

FSDS-R

Screening questionnaire for measuring personal distress related to sex in women with female sexual dysfunction:

How often do you experience:	never 0	rarely 1	occasionally 2	frequently 3	Always 4
1. Anxiety about sexual life	0	1	2	3	4
2. Dissatisfaction with your sexual relationship	0	1	2	3	4
3. Feelings of guilt due to sexual difficulties	0	1	2	3	4
4. Frustration with your sexual problems	0	1	2	3	4
5. Stress about sex	0	1	2	3	4
6. Feeling of inferiority due to sexual problems	0	1	2	3	4
7. Concern about sex	0	1	2	3	4
8. Sexual inadequacy	0	1	2	3	4
9. Regrets about your sexuality	0	1	2	3	4
10. Embarrassment about sexual problems	0	1	2	3	4
11. Dissatisfied with your sex life	0	1	2	3	4
12. Anger about your sex life	0	1	2	3	4
13. Annoyed by low desire sexual.	0	1	2	3	4
